# Risk and Ethical Concerns of Hunting Male Elephant: Behavioural and Physiological Assays of the Remaining Elephants

**DOI:** 10.1371/journal.pone.0002417

**Published:** 2008-06-18

**Authors:** Tarryne Burke, Bruce Page, Gus Van Dyk, Josh Millspaugh, Rob Slotow

**Affiliations:** 1 Amarula Elephant Research Programme, School of Biological and Conservation Sciences, University of KwaZulu-Natal, Durban, South Africa; 2 Department of Fisheries and Wildlife Sciences, University of Missouri, Columbia, Missouri, United States of America; University of Kent, United Kingdom

## Abstract

**Background:**

Hunting of male African elephants may pose ethical and risk concerns, particularly given their status as a charismatic species of high touristic value, yet which are capable of both killing people and damaging infrastructure.

**Methodology/Principal Findings:**

We quantified the effect of hunts of male elephants on (1) risk of attack or damage (11 hunts), and (2) behavioural (movement dynamics) and physiological (stress hormone metabolite concentrations) responses (4 hunts) in Pilanesberg National Park. For eleven hunts, there were no subsequent attacks on people or infrastructure, and elephants did not break out of the fenced reserve. For three focal hunts, there was an initial flight response by bulls present at the hunting site, but their movements stabilised the day after the hunt event. Animals not present at the hunt (both bulls and herds) did not show movement responses. Physiologically, hunting elephant bulls increased faecal stress hormone levels (corticosterone metabolites) in both those bulls that were present at the hunts (for up to four days post-hunt) and in the broader bull and breeding herd population (for up to one month post-hunt).

**Conclusions/Significance:**

As all responses were relatively minor, hunting male elephants is ethically acceptable when considering effects on the remaining elephant population; however bulls should be hunted when alone. Hunting is feasible in relatively small enclosed reserves without major risk of attack, damage, or breakout. Physiological stress assays were more effective than behavioural responses in detecting effects of human intervention. Similar studies should evaluate intervention consequences, inform and improve best practice, and should be widely applied by management agencies.

## Introduction

Successful lobbying against hunting practices by animal-welfare and animal-rights groups [1] as well as limited data regarding the potentially negative long-term effects of direct intervention activities [2] has generated public concern around effects of management intervention on animal species. This is especially true for species that hold special appeal to humans in terms of their charisma, size, danger and drama associated with them [3], [4], and even more so if these same species appear on rare and/or endangered lists [3]. Management interventions that are perceived negatively also have the potential to reduce public appeal, and hence tourism, not only to specific reserves but to protected areas in general. In Africa in particular, many protected areas are dependent on the revenue generated by non-consumptive tourism [5], which therefore has implications for the continuation and very survival of its protected areas.

African elephants (*Loxodonta africana*) hold special appeal to humans not only due to their high tourist value as one of the ‘Big 5’ species, but also because they are social animals that form strong and long-lasting bonds between individuals [6], resulting in humans developing a strong sense of empathy for them. Further, large animals such as elephants pose a potential danger to human life, to infrastructure, and can break out of the fenced areas in which they occur. Major management intervention such as hunting may elicit unpredictable, dangerous responses, and management agencies have to minimise such risks. From both ethical and conservation perspectives, it is therefore essential to quantify the effects of direct human intervention on animal populations.

Animal physiological stress can be measured non-invasively through the measurement of glucocorticoid metabolites (i.e., cortisol or corticosterone) in faeces across a variety of taxa (e.g. 7. 8. [9]). This allows for an accurate assessment of stress without the bias of capture- or disturbance-induced increases in glucocorticoid levels (e.g. [8], [10], [11]). Stress assessment in wildlife serves as a forewarning of possible deleterious impacts from human activities [12].

We therefore aimed to determine the effect of direct human intervention on elephants in a small reserve through behavioural observations and the quantification of the physiological stress responses using faecal hormone metabolite concentrations. Specifically, we determined whether, in response to hunting, elephant showed changes in their (1) movement patterns, (2) grouping patterns (among the breeding herds), and (3) physiological stress levels. Further, we assessed whether physiological stress assays and behavioural observations corresponded. Most importantly, we assessed for extreme responses on the part of the elephants such as attacks on people, infrastructure, or breaking out from the reserve.

## Results

### ‘Major’ Events

BE01 (BE = Bull elephant) was hunted in June 1996, was wounded by the client, and followed-up by the support-team. He charged, and was shot by the Professional Hunter, but managed to kill the Professional Hunter before collapsing. Apart from this, no other major incidents occurred as a result of any hunts, either among the remaining bulls or the breeding herds. Elephants did not break out of the Park or cause damage to infrastructure, and were not responsible for any tourist-related incidents relative to any of the eleven bull hunt events that occurred in the Park.

### Responses of individuals present at the hunt of the targeted bull

Details of all behavioural responses are provided in the supplemental materials (see Text S1 for descriptions and rate of movement; Figure S1 for movement dynamics), and the key results across all hunts are summarized here. For the three hunt events where bulls were present, in all cases the bulls rapidly moved away from the hunt site, and displaced a relatively large distance. In general, the bulls that were present increased their distance from the hunt site in the ten days following the hunt relative to the ten day prior to the hunt. There was no pattern for a change in the direction of movement of bulls associated with the hunted animal in the ten days before relative to after the hunts. For one hunt, the two bulls present at the hunt increased their daily displacement rate after the hunt, but this did not change for bulls present at the two other hunts. There was no clear pattern of shifting home range for those individuals present at the hunt event.

**Figure 1:**
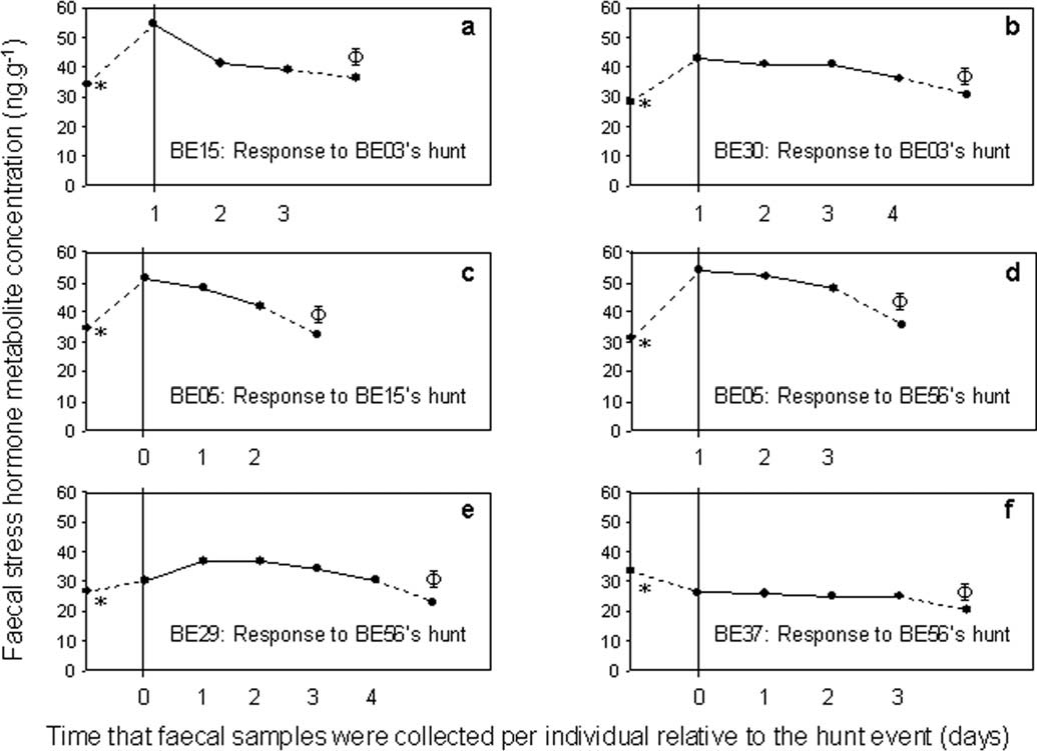
The effect of bull hunts on other bulls present at the hunt. We present the faecal stress hormone metabolite concentrations of the six individual bulls that were in association with the respective targeted bulls at the time of their hunts. ‘*’ represents the individual's baseline stress hormone concentrations in the one-month period before the respective hunt event; ‘Φ’ represents the individual's baseline stress hormone concentrations in the one-month period after the respective hunt event. Vertical lines represent the first day relative to the hunt events when faecal samples from individuals were collected (0 = day of hunt, 1 = one day after hunt etc.). Up to four days passed after the hunt events before the individuals' faecal stress hormone metabolite concentrations were comparable with their initial (one month before hunt) baseline values. There was a significant increase between the maximum faecal stress hormone metabolite concentration and the respective ‘before hunt’ baseline faecal stress hormone metabolite concentrations (Wilcoxon signed-ranks test: T = −2.20, N = 6, P = 0.028).

Physiologically, the maximum time taken for those bulls that were in the presence of the hunted bull to return to levels of faecal stress hormone metabolite concentrations similar to their baseline levels in the one-month period prior to the hunt event was four days, i.e. there was a clear, relatively short, physiological response (Figure 1). To assess a slightly longer physiological response, we used a before-after control design. However, we factored out the four days after the hunt event to remove the extreme response indicated above. We thus compared the average stress levels over a one-month period, and compared the one month before the hunt to the one month starting five days after the hunt. There was no significant difference between the ‘one-month before hunt’ average faecal stress hormone metabolite concentration and the ‘one-month after hunt’ average faecal stress hormone metabolite concentration (Wilcoxon signed-ranks test: T = −0.11, N = 6, P = 0.92). There was a significant increase in the six individuals' (that were present at the hunt events) average maximum (of the three maximum values) faecal stress hormone metabolite concentration in the four-day period following the hunt relative to their ‘one-month before hunt’ baseline metabolite concentration values (Wilcoxon signed-ranks test: T = −2.20, N = 6, P = 0.028) (Figure 1). The hunt events therefore induced significant physiological stress responses in those individuals that were present at the actual hunting of the targeted individual. The behavioural observations of these same bulls showed that they exhibited a ‘flight’ response to the hunt events, but that their movements stabilised after one day following the hunt. Thus the physiological stress response was more long-lived than the behavioural ‘flight’ response.

### Response of individuals not in close proximity to the hunt of the targeted bull

None of the four hunt events induced significant changes in the direction of movement relative to the respective hunt sites for any of the bulls or breeding herds not in close proximity to the hunt. (Table 1). Of the total number of bulls analysed, only 11% were found to move significantly further and 7% to move significantly closer to the respective hunt sites in the ten-day period following the hunt events (Table 1). For the breeding herds, 45% moved significantly further and 7% significantly closer to the respective hunt sites in the ten-day period following the hunt. (Table 1). Forty three percent of all the bulls analysed relative to the four hunt events showed an increase in their displacement rates following the hunts (Table 1); thus approximately half of the bulls increased and half decreased their displacement rates following bull hunt events. Similarly, 45% of all of the breeding herds analysed showed an increase in their displacement rates following the hunt events (Table 1); thus approximately half of the herds increased and half decreased their displacement rates in response to bull hunt events.

**Table 1:** Effects of four hunts on the movement dynamics of bulls and breeding herds not present at the hunts of the targeted bulls.

Sex	Hunt	Number assessed	Move further	Move closer	Faster rate	Slower rate	Increase Range	Decrease Range
Bulls	BE03	4	0	2	1	3	2	2
	BE15	7	1	0	1	6	4	3
	BE56	7	1	0	3	4	1	6
	BE28	10	1	0	6	4	5	5
Breeding Herds	BE03	4	3	0	3	1	1	3
	BE15	4	2	0	2	2	1	3
	BE56	10	8	0	5	5	7	3
	BE28	11	0	2	7	4	4	7

Data are the number of bulls or breeding herds that responded out of those for which we had data for that particular hunt (number assessed).

Only those bulls and breeding herds that showed significant changes in response variables (Mann-Whitney: p<0.05) are presented (i.e., numbers in some columns do not add up to number assessed). No animals showed a directional shift towards or away from the hunts, and those response variables are not included in the table.

There was no significant change in the bulls' core home range sizes in response to the hunt in three cases (Wilcoxon signed-ranks test: Hunt of BE03: T = −0.31, N = 6, P = 0.75; Hunt of BE15: T = −0.56, N = 9, P = 0.58; BE28: T = −0.36, N = 10, P = 0.72), while there was a significant increase in the bulls' core home range sizes in the month after the hunt of BE56 occurred (T = −2.09, N = 10, P = 0.037) (Table 2).

**Table 2:** The effects of the four hunts on bulls' and breeding herds' ranging from one month before to one month after the respective hunt events

Sex	Hunt	N	Average (%) Overlap in Total Range after vs before a	Core Range Average Increase Factor	Individuals with increase in core range size (%) b
Bulls	BE03	6	83.5	1.7	67
Bulls	BE56	10	70.2	7.3	70
Bulls	BE15	9	66.4	2.3	56
Bulls	BE28	10	61.8	3.1	50
Breeding herds	BE03	4	81.8	2.6	75
Breeding herds	BE56	6	79.7	5.6	67
Breeding herds	BE15	5	72.6	2.5	20
Breeding herds	BE28	7	42.3	2.8	14

a Percent overlap of the total range (i.e. area enclosed by 95% Kernel) after hunts to the range before the hunts.

b Percent of individuals whose core home ranges (i.e. areas enclosed by the 50% Kernel) increased from ‘before’ to ‘after’ the hunt events (N is given in third column).

There was no significant change in the respective breeding herds' core home range sizes in response to the hunt in three cases (Wilcoxon signed-ranks test: BE03: T = −1.46, N = 4, P = 0.14; BE15: T = −1.75, N = 5, P = 0.08; BE28: T = −1.42, N = 11, P = 0.16) (Table 2). As in the case of the bulls, there was a significant increase in the breeding herds' core home range sizes in response to the hunt of BE56 (T = −2.29, N = 10, P = 0.022) (Table 2).

For the animals not present at the hunt, we integrated the analysis of behavioural responses using a combined probabilities test. Bull hunt events did not have substantial impacts on the remaining elephants' movement dynamics, bulls' and breeding herds' distances from the hunt sites, direction of movements relative to the hunt sites, displacement rates, core home range sizes, and, for the breeding herds, the fission and fusion dynamics (P>0.05 for all tests) (Table 3).

**Table 3:** Overall effects of the four bull hunts on all the adult bulls and independent breeding herds analysed using combined probabilities tests (Sokal and Rohlf, 1981).

Sex	Response	Statistical Test	−2∑ln P	d.f.	P
Bulls	Distance from hunt site	Mann-Whitney	40.55	70 a	>0.995
	Direction of movement	Sign	31.40	70 a	>0.999
	Rate of movement	Wilcoxon	3.72	8 b	>0.75
	Core home range size	Wilcoxon	3.03	8 b	>0.90
Cows	Distance from hunt site	Mann-Whitney	63.16	60 c	>0.25
	Direction of movement	Sign	43.48	60 c	>0.90
	Rate of movement	Wilcoxon	2.34	8 b	>0.95
	Core home range size	Wilcoxon	0.84	8 b	>0.999
	Fission/fusion	Wilcoxon	1.15	8 b	>0.995

a A total of 35 individual bulls were analysed across all four hunts, giving a degrees of freedom value of number of tests = 70.

b Bulls and breeding herds were analysed collectively for the four hunts, giving a degrees of freedom value of number of tests = 8.

c A total of 30 independent breeding herds were analysed across all four hunts, giving a degrees of freedom value of number of tests = 60

**Figure 2:**
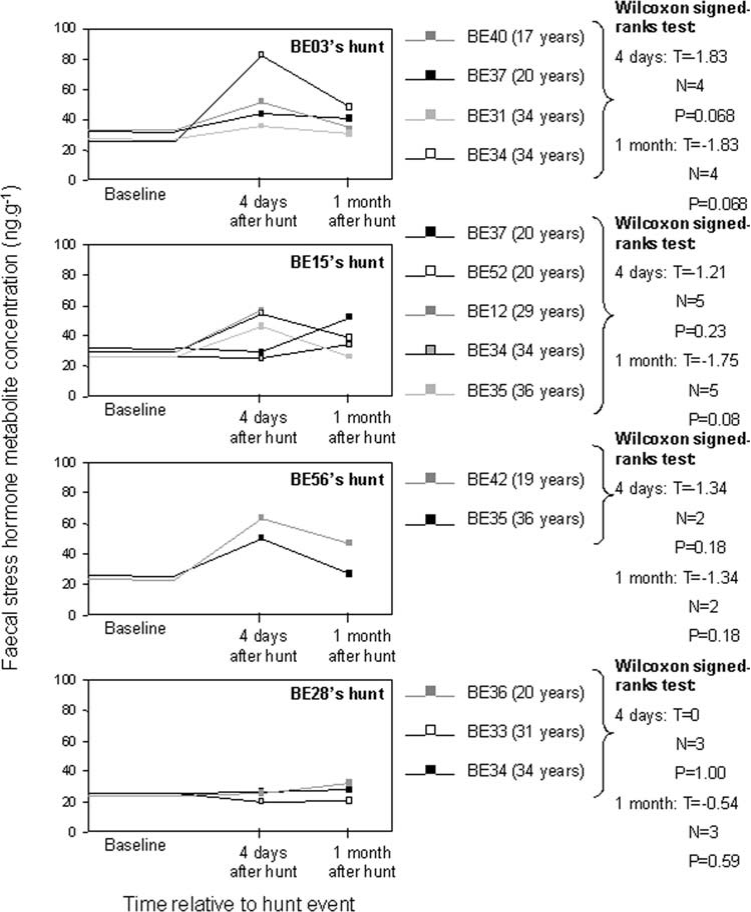
The effect of bull hunts on bulls not present at the hunt. We present the faecal stress hormone metabolite concentrations of individual bulls not associating with the targeted bulls at the time of their hunts. There was no significant increase between baseline: four day and baseline: one month average stress levels of individuals (Wilcoxon signed-ranks test: P>0.05 for all). When individuals were combined, there was a significant increase in baseline: four day and baseline: one month average stress levels (Wilcoxon signed-ranks test: P<0.05 for both).

For individuals not associated with the hunted animal, average faecal stress hormone metabolite concentrations increased significantly relative to their respective baseline values in both the four-day (Wilcoxon signed-ranks test: T = −2.29, N = 10, P = 0.022) and the one-month (Wilcoxon signed-ranks test: T = −0.29, N = 10, P = 0.026) periods following the four hunt events (Figure 2) for the data pooled (averaged) for each of the individuals involved in more than one hunt. Individually, 11 of 14 bulls showed an increase in their average faecal stress hormone metabolite concentrations in the four-day period following the hunt events relative to their baseline faecal stress hormone metabolite levels, and 12 of these bulls experienced increased average levels of faecal stress hormone metabolites relative to their baseline stress levels in the one-month period following the four hunt events (Figure 2).

The individual bulls' faecal stress hormone metabolite concentrations were higher during the four-day post-hunt period as opposed to the one-month post-hunt period for BE03's, BE15's and BE56's hunts (Figure 2). This can be explained in terms of the fact that there were other bulls associating with these targeted individuals at the time of them being hunted. It is likely that these non-hunted bulls emitted distress vocalisations for some period of time following the hunt event. Since elephant vocalisations can be transmitted over relatively long distances (e.g. [13], [14]), it is possible that these bulls' distress calls were received by the remaining bull population, resulting in a general increase in stress being induced. There were no real effects on other bulls from BE28's hunt (Figure 2).

All of the bulls for which faeces were collected experienced elevated stress hormone concentrations in response to the four hunting events. This is in contrast to the behavioural observations, where only 11% of the bulls observed were found to significantly change their movement dynamics in terms of their distances from the respective hunt sites in the ten-day period following the hunt events, and only half of them increased their core home range sizes in the one-month period following the hunt events. Thus behavioural observations alone did not comprehensively quantify the effects of hunting individuals on the remaining bull population as behavioural responses relative to the hunt events were only observed for some individuals whereas increased physiological stress was detected for all individuals.

**Figure 3:**
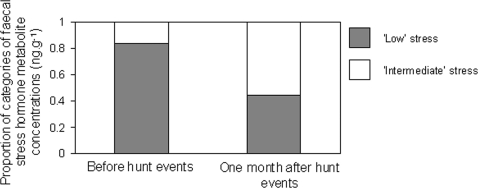
The effect of bull hunts on breeding herds. We present stress levels ‘before’ and ‘one month after’ the bull hunts as adult females' categorised faecal stress hormone metabolite concentrations (‘low’ = 6.3–40.67 ng.g^−1^ and ‘intermediate’ = 40.68–75.05 ng.g^−1^; no ‘high’ concentrations were recorded).

During the ‘before’ hunt periods, the adult females had higher frequencies of ‘low’ stress levels while in the period following the hunt adult females had higher frequencies of ‘intermediate’ stress levels (Figure 3). This indicated a general physiological stress response to the hunts. However, analyses of the breeding herds' movement dynamics showed no significant changes in response to the hunt events.

We observed twelve herds before and after each hunt, and assessed a fission/fusion response by measuring the percentage of time that they spent with other herds (fusion) or alone (fission). Breeding herds were found significantly more on their own as opposed to in groups of herds in the ten-day periods following the hunt events (Wilcoxon signed-ranks test: P<0.05 for all) (Table 4).

**Table 4:** The effect of the four hunts on the independent breeding herds' fission and fusion dynamics in the ten-day period before and after each hunt event.

ID	Hunt							
	BE03		BE15		BE56		BE28	
	Before (%)	After (%)	Before (%)	After (%)	Before (%)	After (%)	Before (%)	After (%)
CE01	0	0	100	80	0	100	100	100
CE02	0	25	0	0	0	0	0	7
CE03	0	0	40	80	0	0	12	17
CE07	0	33	0	75	12	0	0	2
CE17	0	0	0	0	24	100	67	11
CE19	0	50	0	0	0	0	0	0
CE20	20	60	0	0	33	44	0	0
CE32	0	0	100	100	100	25	100	33
CE54	0	0	0	0	0	8	56	0
CE56	0	0	0	0	6	14	67	17
CE57	0	0	0	0	32	0	40	0
CE59	0	0	0	0	40	33	0	75

Data are percent that each herd was seen with other herds out of the total number of sightings for that herd in the given time period (larger values indicate greater association, 0 indicates that the herd was seen, but was always on its own).

## Discussion

In response to short duration hunting events, wildlife often exhibit short-term behavioural responses; however, corresponding physiological responses have not been measured. Behavioural responses by wildlife to hunting activity includes directed movement away from hunters [15], changes in diet [16], distributional shifts [17], greater use of vegetative cover [15] or shifts in core area use within the home range [18]. Behavioural responses to hunting might also be related to prior experiences. For example, elk (*Cervus elaphus*) became less tolerant of hunter activity later in the hunting season, resulting in more extreme movements as the season progressed [15]. Despite a consistent behavioural response, we are not aware of any study documenting a physiological stress response to hunting. For example, in Spain, Dalmau et al. [19] did not find a relationship between hunting activity and stress in Pyrenean chamois (*Rupicapra pyrenaica pyrenaica*), as measured by faecal glucocorticoid metabolites. Despite the behavioural responses noted above, Millspaugh et al. [8] did not observe a correlation between hunting activity and faecal glucocorticoid metabolites in elk. Extending hunting hours did not increase corticosterone levels in mourning doves (*Zenaida macroura*) when compared with baseline values [20]. These studies of physiological stress collectively point to the importance of physical and environmental (e.g., weather) stressors. In contrast, we hypothesize that stress in elephants, which have a complex social system, might be heavily influenced by psychosocial stressors that result from hunting activity. Social vertebrates often exhibit increases in stress due to psychosocial stressors [21].

Many private reserves and protected areas in southern and eastern Africa make use of trophy hunting for income generation, and as a means of both eliminating ‘problem’ elephant bulls [22], [23] and manipulating the population's linear dominance hierarchy [24]. This is the first detailed study quantifying the effects of bull hunting on the remaining elephants. One might expect, particularly in a relatively small, confined population, that such direct management intervention would significantly affect the remaining elephants. However, only one major reaction occurred, as a result of a poor kill, and the only notable minor behavioural effects were short-term (one day) ‘flight’ responses by bulls present at the hunt. Physiologically, the induced stress response (both short and longer-term) manifested throughout the population, indicating communication amongst individuals, and may represent the reaction of elephants to each other's suffering (see [25]). The response from those individuals that were present at the hunts of the targeted bulls may have been transmitted by means of a ‘domino-effect’ throughout the remaining bull population. Interestingly, our results indicate transmission of stress events from bulls to cows.

Limited behavioural studies investigating the specific effects of direct human impact on elephants have been conducted. Poaching pressure leads to increasing group size [26], and ‘migration’ and aggregation into protected areas [27]. Culling causes disturbance [28], with elephant from a culled population shifting to drink more at night [22]. Four out of the 10 collared female elephants that were within 7 km of a culled group undertook extreme direct movements of 23, 25, and 30 km overnight or within two days of the cull, and which took them out of their then pre-determined range [29].

The results of this study can be applied by elephant managers in small reserves, and may contribute to future debates regarding the implementation of elephant population control through selective bull hunting (see [30] for a treatment of the broader ethical question of hunting elephants). We conclude that hunting bulls in the manner and frequency described here will have no major effect on the remaining elephant population. Despite the increase in faecal glucocorticoid metabolites, the stress response in elephants was short-lived and, in our opinion, not detrimental (the peaks being lower than that shown to natural extreme stressors such as transport or extreme, loud, noises including thunderstorms [9]). However, bulls should preferably not be hunted when they are in musth as they are more aggressive and unpredictable than usual [31], [32]. Because of observed stress responses of bulls present at the hunt, best practice to reduce unnecessary stress indicates that bulls should only be hunted when they are alone. We also recommend that sufficient time (in this study found to be one month) between direct disturbance events be allowed to reduce the possibility of chronic stress (i.e. cumulative effects which were not assessed in this study) in the population. However, we also caution that the effects of disruption of the dominance hierarchy, through hunting, on stress levels, movements and other behaviour has not been investigated, and may, in sustained hunting situations, be significant.

## Materials and Methods

Pilanesberg National Park (25°8′S–25°22′S; 26°57′E–27°13′E, 560 km^2^) is located in the North West Province of South Africa. The area comprises predominantly hilly terrain and falls within the transition zone between the Kalahari Thornveld in the west and the Bushveld in the east. The habitat comprises *Acacia* and broad-leaf bushveld, which ranges from closed thickets to open grasslands. The general vegetation type is classified as sourveld [33]. One major river system runs through the centre of the Park, and numerous non-perennial tributaries and streams and several small dams are scattered throughout. Rainfall occurs in summer, and is approximately 630 mm per annum. Temperatures range from a minimum of 1–5°C in winter to a mean maximum of 28–31°C in summer. The park border fence is electrified and provides an effective barrier to elephants (no breakouts to date).

Elephants were introduced, mainly from Kruger National Park between 1981 and 1998 [34]. In 1998, when the population was comprehensively identified to the individual level for the first time, there were 93 elephant, including 17 individually recognizable independent males. These were males that had left the female groups, and were consistently alone or with other males. These ranged in age from 18 to 25 years old. There were also six older males (up to 35 years old) introduced to solve the rhino-killing problem (see [34]). By March 2002, there were 163 elephant, including 39 adult bulls between 12 and 40 years old, and 12 known breeding herds.

Pilanesberg National Park leases out a hunting concession on an annual basis. The Board and Park management are responsible for formulating a hunting quota per species, which is revised annually based on species' abundance and the Park's objectives. Two elephant hunts are usually sold per annum, where each hunt generates an average of $10 000 (approximately R70 000). This revenue is fed back into the Park management.

In 1996, elephant bulls were identified as a major source of both black and white rhinoceros mortality in the Park [24]. The Park controlled these ‘problem animals’ by hunting two known culprits [34], as well as five problem animals (chasing rhino or damaging vehicles or infrastructure) between 1996 and 2001.

A total of eleven bulls were hunted from 1996 through 2003, with the last four hunts being intensively studied. (1) BE03 (prefix ‘BE’ denotes bull and ‘CE’ cow elephants, with a unique individual numerical code following) was hunted on the 16 April 2002. BE15 and BE30 were with him at the time of the hunt. (2) BE15 was hunted on the 30 July 2002. BE05 was with him at the time of the hunt. BE43 and CE01's herd were within 2 km of the hunt. (3) BE56 was hunted on the 9 May 2003. BE05, BE29 and BE37 were with him at the time of the hunt. (4) BE28 was hunted on the 7 August 2003. He was alone at the time of the hunt. A professional hunter, a Park representative, and a ground crew of at least four individuals always accompanied the client, and hunts took place from early to mid-morning. In ten of the 11 hunts, the bull was killed cleanly and went down almost immediately. The exception was a musth bull hunted in 1996, who was wounded by the client and killed by the professional hunter. For the four intensive hunts, targeted bulls were followed intensively in the week leading up to a hunt, and we recorded the bull's locations, associations with other bulls and breeding herds, and daily displacement rate.

### Behavioural observations

The effects of the hunts on the elephant population were divided into ‘major’ and ‘minor’ events: ‘Major’ events included elephants breaking out of the Park, causing damage to infrastructure, and being responsible for tourist-related incidents (e.g. damaging vehicles, increased aggression etc.) associated with the hunt events. ‘Minor’ events referred to less obvious responses of the remaining elephants to the hunt events. Since movement is often the easiest parameter to measure when assessing the responses of animals to disturbance, we recorded the elephants' movement dynamics in response to the hunt events. The effect of the hunt on the movement dynamics of the remaining elephant population was divided into the short- and longer-term, with the short-term referring to the ten-day period before and after the hunt and the longer-term being the period one month before and one month after the hunt. Only those bulls and individual breeding herds that were located at least ten times each in the month before and after the hunt were used in the analysis. No unusually different behaviour was noted in any of the other animals that were excluded from the analysis for lack of sufficient sample sizes.

All elephants were identified using distinctive markings. Each of the matriarchs of the twelve herds were radio-collared with VHF transmitters, which facilitated locating them. A concerted effort was made to locate as many individual elephants as possible each day, and particularly for uncollared males, tourist guides criss-crossing the reserve (and who were familiar with the identities of individual males) helped with locations. All locations were confirmed by us. We searched intensively to find individuals that were with the hunted animal, or close-by. Each individual bull's and breeding herd's locations for the period ten days before and ten days after the hunt were mapped using Animal Movement extension in ArcView 3.1 (ESRI) [35], and a polyline indicating the direction moved between each point was created. The distance between each location point and the hunt site was calculated in ArcView and converted to km. Distances before and after each hunt event were contrasted using the Mann-Whitney U-test, with each individual representing independent data points.

The direction of movement relative to the hunt site was calculated manually using printouts of each individual's movements from ArcView. The direction moved between two points (see Figure 1 x-axis for time interval between points for each individual per hunt) was determined by using the straight line between the first point and the hunt site as a reference line (i.e. 0° line on the protractor) and determining the angle between the reference line and the straight line drawn between the first and second points. This was done on a 0–180° scale, with 0–90° indicating movement towards the hunt, and 90–180° indicating movement away from the hunt. Each value was then assigned as ‘positive’ (towards the hunt) or ‘negative’ (away from the hunt). Changes in direction moved before and after hunts were assessed using sign tests, with the average each individual representing independent (paired) data points. The direction data were then plotted against the distance data to illustrate the direct (short-term) effect of the hunt on the movement dynamics of each individual bull or breeding herd.

The displacement rate of each bull and breeding herd in the ten-day period before and after each hunt event was determined by calculating the distance between each successive location point in ArcView, and then dividing the distance by the respective time interval. To remove any bias in the analysis for those individuals where more than one location per day was obtained, the first datum recorded per day was used in the calculation of rate of displacement. For the analysis of those bulls that were present at the actual hunting of the targeted individual, their first recorded displacement was their departure from the hunt site. Since only the first location for these bulls was used, the results would only be biased conservatively in favour of them increasing their distance from the respective hunt sites. Because the times of locations for each individual were different, the displacements were calculated and expressed in terms of km.h^−1^. The coefficient of variation (CV) [36] was calculated for each individual bull and breeding herd's displacement rate before and after the respective hunt events, with a higher CV indicating more erratic displacement rates. Displacement rates before and after each hunt event were contrasted using the Mann-Whitney U-test, with average values for each individual representing independent data points.

To determine whether the breeding herds showed a ‘fission’ or ‘fusion’ (i.e. whether the herds came together or dispersed) response following the hunts, the number of matriarchs (where one matriarch indicates the presence of one herd) seen together in the ten-day period before the respective hunt and in the ten-day period after the hunt was compared. We calculated the total number of sightings of each herd in the ten-day period before and after the hunt, and dividing these sightings into whether the herd was alone or with other herds. The frequency of being with other herds was calculated, so that each herd had one value representing their ‘grouping’ tendency before and one value representing their ‘grouping’ tendency after the hunt. Thus each independent datum represented a single breeding herd. The percentage sightings with other herds (fusion) was contrasted before and after each hunt event using the Wilcoxon test, with each herd representing independent (paired) data points.

Each individual bull's and breeding herd's locations (using the first location per day) for the period of one month before and one month after the hunt were mapped in ArcView (ESRI). Separate fixed kernel home ranges [35], using the 95 % and 50 % probability contours, were plotted for the month before and after the hunt. These were then overlayed, and the percentage area overlay from the ‘before hunt’ home range and the ‘after hunt’ home range was computed to determine whether a shift in home range (long-term effect) had occurred subsequent to the hunt. The core home range (area enclosed by the 50 % probability contour) from before and after the hunt were also determined, and represented as a factor increasing or decreasing relative to the ‘before hunt’ core home range area. Range sizes were contrasted before and after each hunt event using the Wilcoxon test, with each individual representing independent (paired) data points.

Data were not normally distributed (Kolmogorov-Smirnov test: P<0.05), and thus non-parametric tests (Sign test, Mann-Whitney U test, Wilcoxon signed-ranks test) were used in the statistical analyses.

The overall patterns of behavioural response was tested using the Combined Probabilities Test. The ‘combined probabilities’ analysis allows for separate significance tests on different data sets that test the same scientific hypothesis to be combined and analysed collectively [37]. The probability values obtained from the four separate analyses conducted for each of the individual bulls and the five analysis for each of the independent breeding herds relative to the hunt events (i.e. distance from hunt site, direction of movement relative to hunt site, displacement rate, core home range sizes and fission and fusion responses (breeding herds only)) (see above for detail) were tested using combined probabilities [37]. These analyses reflect the more robust assessments of behavioural responses as the problem of pseudoreplication, which may be present in the individual based analyses, is largely negated.

### Physiological stress response

Dung samples were collected throughout the study period from March 2002 to July 2003. The protocol for collecting, storing and processing the samples and the extraction of cortisol from the samples is extensively described elsewhere [11], [38].

Upon collection, each sample was allocated a unique numerical code and the date, time of sample collection, bolus measurements (top diameter, bottom diameter and length), location (GPS co-ordinates and name of road nearest to sample), and the identification of the elephant known to have defecated were recorded. Some samples were collected without knowing which individual had deposited them. These samples were ‘sexed’ and aged by (1) referring to the spoor around the sample and (2) using the diameter of the bolus where adult (>15 yrs) bulls' boluses were generally found to have a minimum diameter of 12 cm, while adult (>15 yrs) cows' boluses generally had a minimum diameter of 10 cm, sub-adults (6–15 years) a minimum diameter of 6–9 cm, and juveniles (less than 6 years) a minimum diameter of less than 6 cm , based on samples from known individuals (pers. obs.).

The actual time of sample collection was corrected per sample based on the estimated age of the sample. Thus if the sample was estimated as being 5 h old, then 5 h were subtracted from the ‘collected’ time to give a ‘corrected’ time.

A ‘lag-time’ of 36 h (the time taken for a ‘stressful’ event to be maximally detected in African elephant faeces [7], was used to correlate specific events with the faecal stress hormone metabolites present in the dung samples. We subtracted 36 h from the ‘corrected time’ of each sample to give the actual time at which the stress occurred.

Samples were categorised into ‘low’, ‘intermediate’ and ‘high’ stress by determining the total range of faecal stress hormone metabolite concentrations from all samples collected (6.3–109.43 ng.g^−1^), the physiological stress response of elephants [7], [9], and dividing them into three parts. Thus samples with 6.3–40.67ng.g^−1^ stress hormone metabolite concentrations = ‘low’ stress, samples with 40.68–75.05ng.g^−1^ stress hormone metabolite concentrations = ‘intermediate’ stress, and samples with 75.06–109.43ng.g^−1^ stress hormone metabolite concentrations = ‘high’ stress. The basis for these categorisations is supported by the ACTH challenge results [7], [9].

There were a total of six bulls that were associating with the respective hunted bulls (within 10 m of the targeted bull) at the time of the hunt events. Baseline faecal stress hormone metabolite concentrations were obtained for each of these six bulls by averaging their respective ‘low’ stress level faecal metabolite concentrations from one month before the hunt event (only the ‘low’ stress level category was used in order to account for individual variation in the stress response (e.g. [39], [40], [41]). The time taken for each of these six bulls to return to their respective baseline faecal stress hormone metabolite concentrations was determined by plotting their actual stress level concentrations against time (in terms of days after hunt event). The baseline faecal stress hormone metabolite concentrations for these six bulls after the hunt events was calculated by averaging their ‘low’ stress level faecal metabolite concentrations for one month after their metabolite concentrations had returned to their former baseline levels. The baseline faecal stress hormone metabolite concentrations of the individual bulls in the one-month before the hunt were tested for significant differences with (i) their baseline faecal stress hormone metabolite concentrations in over one-month after the hunt (i.e. controlling for change over time), and (ii) with the average of the maximum three faecal stress hormone metabolite concentration values obtained during the time that it took for each of the individuals to return to their baseline faecal stress hormone metabolite concentrations after the hunt. The Wilcoxon signed-ranks test was used.

Sufficient faecal samples in the one-month period following each hunt event were collected from fourteen individually identified adult bulls from those not associating with the targeted bull at the time of his hunt. Four of these bulls were ‘replicates’ in that sufficient samples were collected from each of them relative to more than one hunt. The Analyses were conducted for a four-day (short term) and a 5–30 day (long-term) period following each hunt event. The average of the three maximum values obtained for each individual was used. Baseline faecal stress hormone metabolite concentrations for each individual were calculated using the remaining samples in the ‘low’ stress level category collected throughout the broader study period. The 4 and 5–30 day post-hunt values were tested for significance against the baseline average faecal stress hormone metabolite concentration value using the Wilcoxon signed-ranks test with each individual (paired) representing independent data points.

Insufficient identified faecal samples from individuals within a particular breeding herd precluded using an individual-based analysis for female responses. Instead, the adult (15 years and older, to exclude the possibility of age confounding the results) breeding herd animals' faecal stress hormone samples were combined to give a general breeding herd response to hunt events. This is made possible due to the fact that (1) members of the same herd show synchronous increases in stress hormone metabolites in response to ‘stressful’ events [42, Millspaugh, unpublished data], and (2) the strong social bonds existing between different family groups (e.g. [6]) and the fact that elephants can communicate over relatively long distances (e.g. [13], [14]) allow for the assumption that this synchronicity in stress hormone production will extend throughout the breeding herd population, particularly in a relatively small reserve such as Pilanesberg National Park. Data for one month following each of the four hunts were categorised as being ‘After’ the hunt events. The remaining data were classified as being representative of ‘Before’ the hunt events. The respective samples were classified into ‘low’, ‘intermediate’ and ‘high’ levels of stress. We used log-linear analyses [43] of the different categories (‘Low’, ‘Intermediate’ and ‘High’ stress) before versus after the hunt.

## Supporting Information

Text S1 Supporting Information text file including table for Burke et al.
(0.05 MB DOC)

Figure S1 The effect of bull hunts on movement dynamics. We assess bulls and breeding herds associated with the hunt event in terms of their distance and direction moved relative to the hunt site for the ten-day period before and after the hunt. X_B_ = mean distance to hunt site before hunt. X_A_ = mean distance to hunt site after hunt. Mann-Whitney U tests were used to determine whether significant changes in distance from the hunt site occurred (U and P values displayed). 0-90 0 indicates movement towards the hunt, and 90 0-180 0 indicates movement away from the hunt. Sign tests were used to determine whether significant changes in direction of movement relative to the hunt site occurred (P>0.05 for all, therefore not displayed). Open boxes = observations ten days before hunt events; black boxes = observations ten days after hunt events.
(1.63 MB TIF)
