# Supplementary material for: Risk and Ethical Concerns of Hunting Male Elephant: Behavioural and Physiological Assays of the Remaining Elephants
Source: PLoS One. 2008 Jun 18;3(6):e2417. doi: 10.1371/journal.pone.0002417 (PMC2426916; doi:10.1371/journal.pone.0002417)
Supplement: Text S1 — Supporting Information text file including table for Burke et al. (0.05 MB DOC) [file pone.0002417.s001.doc]

**Supporting Information**

**Detailed behavioral responses of individuals present at the hunt of the targeted bull**

BE15 and BE30 were found associating with BE03 in the four-day period leading up to BE03’s hunt, and were together (feeding within 10 m of each other on a slope) at the time of the hunt event. Once the first shot was fired BE15 and BE30 immediately began running. They moved in an easterly direction over the ridge of the slope that they were on and into the adjacent valley, moving over 1.5 km from the hunt site in the hour after the shots were fired. For the hunt of BE03, there was a significant increase in the distance that BE15 moved relative to the hunt site from the ten-day period before (mean distance from hunt site of sightings before= 1.2 km) compared to the ten-day period after (mean distance = 8.4 km) the hunt occurred (Mann-Whitney U test: U = -2.20, N_before_ = 5, N_after_ = 8, P = 0.028) (Figure S1a). On average BE30 also moved further away from the hunt site in the ten-day period after (mean distance = 7.7 km) relative to before (mean distance = 2 km before hunt) the hunt occurred. However this difference was not statistically significant (Mann-Whitney U test: U = -1.79, N_before_= 6, N_after_ = 6, P = 0.13) (Figure S1b). Neither of the bulls showed any significant change in the direction that they moved relative to the hunt site before (Sign test: P > 0.05) and after (Sign test: P > 0.05) the hunt (Figure S1a-b). Both bulls showed an increase in their displacement rate (km.h^-1^) in the ten-day period after the hunt event (Table 1). BE30’s coefficient of variance (CV) of displacement rate value remained relatively constant whilst BE15’s CV of displacement rate decreased in the ten-day period following the hunt (Table 1).

BE05 was seen with BE15 one day before the hunt of BE15, and these two bulls were located together on the day of BE15’s hunt (never more than 10 m apart). On the day of the hunt, BE43 and CE01’s herd were located just over 2 km away from the hunt site. Immediately the shots were fired, BE05 ran in an easterly direction for approximately 0.5 km. However, approximately 45 min after BE15 had been hunted, BE05 came back to the site, and was seen sniffing the body and touching it with his trunk before moving off in the direction from which he had initially run. The next day, BE05 was located 6.5 km from the hunt site. BE43 immediately moved out of the area, and was located in the central region of the Park on the day after the hunt, over 6.5 km from the hunt site. CE01’s herd was observed feeding just over 1 km from the hunt site 20 min after the hunt. All of the individuals appeared relaxed, with no temporal streaming, and showed no obvious response to the shots being fired. None of the individuals associated with hunt of BE15 showed significant changes in their distances from the hunt site in the ten-day period before compared with the ten-day period after BE15’s hunt (Mann-Whitney U test: BE05: U = -1.70, N_before_ = 4, N_after_ = 7, P = 0.11; BE43: U = -1.71, N_before_ = 7, N_after_ = 5, P = 0.11; CE01: U = 0, N_before_ = 8, N_after_ = 9, P = 1.00) (Figure S1c-d). BE05 was always moving towards the hunt site in the ten-day period before the hunt occurred, he showed no significant change in his direction of movement relative to the hunt site in the ten-day period following BE15’s hunt (Sign test: P > 0.05) (Figure S1c). There was no significant change in the direction that BE43 (Sign test: P > 0.05) or CE01's (Sign test: P > 0.05) herd moved relative to the hunt site before and after the hunt (Figure S1d-e). BE05’s median displacement rate increased (Table 1) whilst BE43’s median displacement rate decreased in the ten-day period following the hunt (Table 1). Both bulls' CV of displacement rate values remained relatively constant (Table 1). CE01’s herd's median displacement rate increased in the ten-day period following BE15’s hunt (Table 1). However, there was no substantial change in their CV of displacement rate.

BE05, BE29, BE37 and BE56 were seen within 15 m of each other on the day of BE56’s hunt. When the shots were fired, BE05, BE29 and BE37 moved off in a northerly direction, to over 1 km away from the site. BE05 and BE37 remained together until four days following the hunt, moving through the valleys in the south-eastern region of the Park (by then just over 7 km from the hunt site). BE29 moved over 10 km overnight, and was seen in the central region of the Park the following day. Two of the three of the bulls that were with BE56 at the time of his hunt showed substantial movements away from the hunt site in the ten-day period following the hunt event (mean distance before hunt: BE05: 8 km; BE29: 0.2 km; BE37: 1.8 km, compared with mean distance after hunt: BE05: 4.9 km; BE29: 7.9 km; BE37: 6.4 km) (Figure S1f-h). However, because there were so few sightings of the bulls in the ten-day period prior to the hunt, the power for statistical analyses was low, resulting in no significant changes in their distances from the hunt site in the ten-day period before compared with the ten-day period after the hunt occurred being detected (Mann-Whitney U test: BE05: U=-1.44, N_before_= 4, N_after_ = 11, P = 0.18; BE29: U=-1.55, N_before_ = 3, N_after_ = 6, P = 0.17; BE37: U=-1.53, N_before_ = 4, N_after_ = 8, P = 0.15) (Figure S1f-h). These three bulls did not show significant changes in their direction of movement relative to the hunt site in the ten-day period before (Sign test: P>0.05) versus after (Sign test: P>0.05) (Figure S1f-h) BE56’s hunt. They did not show substantial changes in either their short-term median or CV of displacement rates in response to BE56’s hunt (Table 1).

The response in ranging of individuals that were with the hunted elephant at the time were as follows. For the hunt of BE03, BE30 showed the greatest percentage overlap (97% overlap) in home range while BE15 showed the smallest overlap(65%). For the hunt of BE15, BE05 showed an overlap of 61%. CE01’s herd, which was in close proximity to the hunt of BE15 (just over 2 km away), showed the greatest percentage overlap in home range size from before to after the hunt (94% overlap). The three bulls that were present at the hunt of BE56, namely BE05, BE29 and BE37, showed percentage overlaps in core home range size of 43%, 63% and 96% respectively. There was thus no clear pattern of shifting home range for those individuals present at the hunt event.

**Table 1.** The effect of three bull hunts on the rates of movement (km.h^-1^) of individuals that were present at the hunt of the targeted bull in the ten-day period before compared with the ten-day period after each hunt event (no individuals were with the fourth bull hunted).

| Ele ID^a^ | BE03’s hunt | | | | BE15’s hunt | | | | BE56’s hunt | | | |
| --- | --- | --- | --- | --- | --- | --- | --- | --- | --- | --- | --- | --- |
|  | Before | | After | | Before | | After | | Before | | After | |
|  | CV^b^ | Med^c^ | CV | Med | CV | Med | CV | Med | CV | Med | CV | Med |
| BE05 |  |  |  |  | 1.02 | 0.06 | 1.43 | 0.61 | 0.9 | 0.14 | 0.89 | 0.12 |
| BE15 | 1.46 | 0.05 | 0.51 | 0.22 |  |  |  |  |  |  |  |  |
| BE29 |  |  |  |  |  |  |  |  | 0.79 | 0.23 | 0.65 | 0.33 |
| BE30 | 1.21 | 0.06 | 1.17 | 0.15 |  |  |  |  |  |  |  |  |
| BE37 |  |  |  |  |  |  |  |  | 0.62 | 0.19 | 0.73 | 0.16 |
| BE43 |  |  |  |  | 0.95 | 0.31 | 0.78 | 0.14 |  |  |  |  |
| CE01 |  |  |  |  | 0.64 | 0.07 | 0.69 | 0.14 |  |  |  |  |

Prefix ‘BE’ refers to bull elephants; prefix ‘CE’ refers to breeding herds.

^b^ CV refers to the ‘coefficient of variance’ and describes the distribution of the rate of movement values, i.e. the greater the CV value, the more variation in that individual’s rate of movement between two points.

^c^ Med. is the median rate of movement.
