# Supplementary figures and images for: Risk and Ethical Concerns of Hunting Male Elephant: Behavioural and Physiological Assays of the Remaining Elephants
Source: PLoS One. 2008 Jun 18;3(6):e2417. doi: 10.1371/journal.pone.0002417 (PMC2426916; doi:10.1371/journal.pone.0002417)

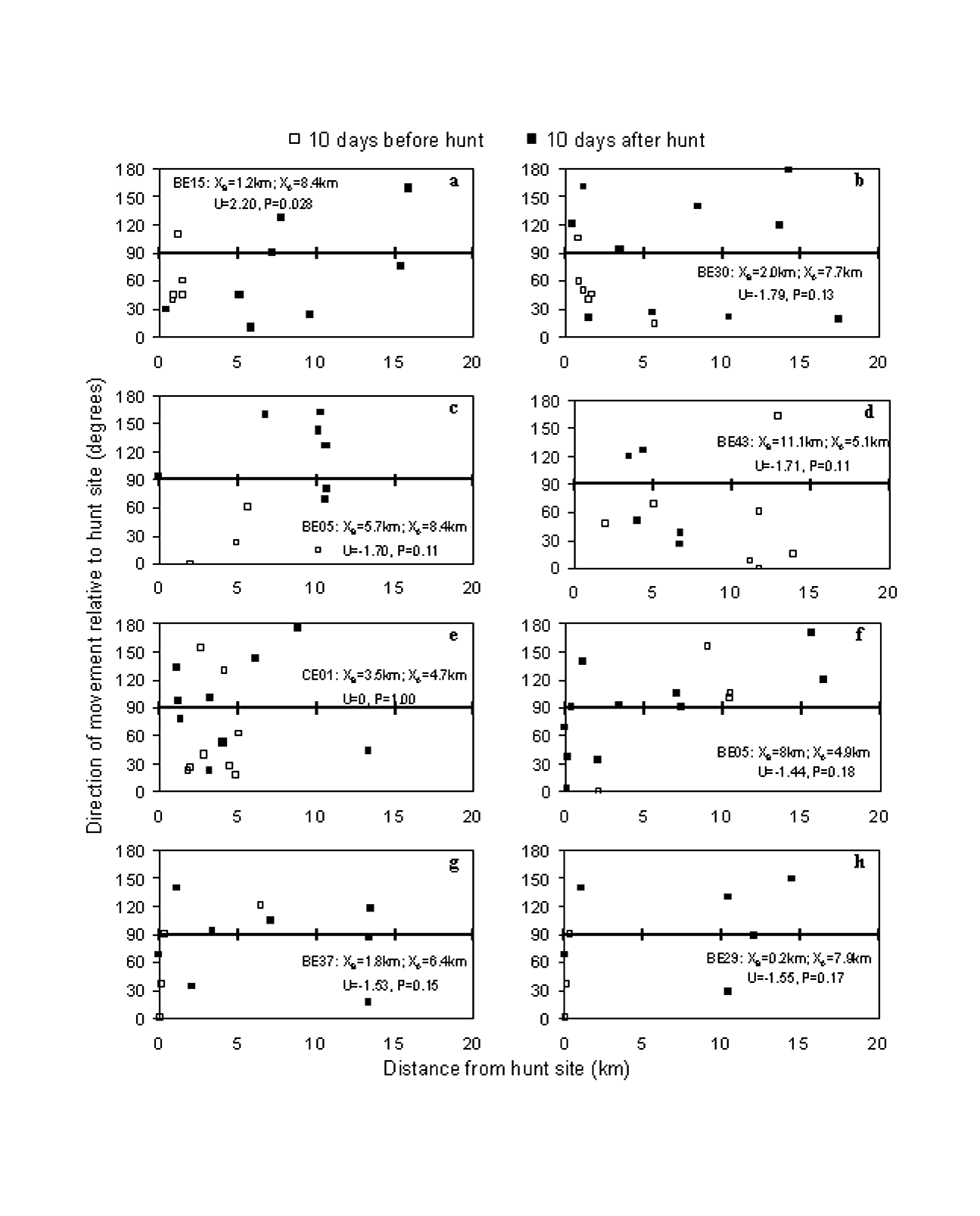

Supplement: Figure S1 — The effect of bull hunts on movement dynamics. We assess bulls and breeding herds associated with the hunt event in terms of their distance and direction moved relative to the hunt site for the ten-day period before and after the hunt. XB = mean distance to hunt site before hunt. XA = mean distance to hunt site after hunt. Mann-Whitney U tests were used to determine whether significant changes in distance from the hunt site occurred (U and P values displayed). 0-90 0 indicates movement towards the hunt, and 90 0-180 0 indicates movement away from the hunt. Sign tests were used to determine whether significant changes in direction of movement relative to the hunt site occurred (P>0.05 for all, therefore not displayed). Open boxes = observations ten days before hunt events; black boxes = observations ten days after hunt events. (1.63 MB TIF) [file pone.0002417.s002.tif]
